# Gold Nanoparticles for Monitoring of Mesenchymal Stem-Cell-Loaded Bioresorbable Polymeric Wraps for Arteriovenous Fistula Maturation

**DOI:** 10.3390/ijms241411754

**Published:** 2023-07-21

**Authors:** Allan John R. Barcena, Joy Vanessa D. Perez, Jossana A. Damasco, Marvin R. Bernardino, Erin Marie D. San Valentin, Carleigh Klusman, Benjamin Martin, Andrea Cortes, Gino Martin Canlas, Huckie C. Del Mundo, Francisco M. Heralde, Rony Avritscher, Natalie Fowlkes, Richard R. Bouchard, Jizhong Cheng, Steven Y. Huang, Marites P. Melancon

**Affiliations:** 1 Department of Interventional Radiology, The University of Texas MD Anderson Cancer Center, Houston, TX 77030, USA; ajbarcena@mdanderson.org (A.J.R.B.); jdperez1@up.edu.ph (J.V.D.P.); damascoja@gmail.com (J.A.D.); mrbernardino@mdanderson.org (M.R.B.); edsan@mdanderson.org (E.M.D.S.V.); benjamin.martin@bcm.edu (B.M.); accortes@mdanderson.org (A.C.); hcd3@rice.edu (H.C.D.M.); rony.avritscher@mdanderson.org (R.A.); syhuang@mdanderson.org (S.Y.H.); 2 College of Medicine, University of the Philippines Manila, Manila 1000, Philippines; fmheralde1@up.edu.ph; 3 School of Medicine, Baylor College of Medicine, Houston, TX 77030, USA; 4 Department of Chemistry, Lamar University, Beaumont, TX 77710, USA; gcanlas@lamar.edu; 5 Department of Veterinary Medicine and Surgery, The University of Texas MD Anderson Cancer Center, Houston, TX 77030, USA; nwfowlkes@mdanderson.org; 6 Department of Imaging Physics, The University of Texas MD Anderson Cancer Center, Houston, TX 77030, USA; rrbouchard@mdanderson.org; 7 Section of Nephrology, Department of Medicine, Selzman Institute for Kidney Health, Baylor College of Medicine, Houston, TX 77030, USA; jizhongc@bcm.edu; 8 The University of Texas MD Anderson Cancer Center UTHealth Houston Graduate School of Biomedical Sciences, Houston, TX 77030, USA

**Keywords:** arteriovenous fistula, computed tomography, end-stage renal disease, gold nanoparticles, mesenchymal stem cells, neointimal hyperplasia, polymer, ultrasonography

## Abstract

Mesenchymal stem cell (MSC)-seeded polymeric perivascular wraps have been shown to enhance arteriovenous fistula (AVF) maturation. However, the wraps’ radiolucency makes their placement and integrity difficult to monitor. Through electrospinning, we infused gold nanoparticles (AuNPs) into polycaprolactone (PCL) wraps to improve their radiopacity and tested whether infusion affects the previously reported beneficial effects of the wraps on the AVF’s outflow vein. Sprague Dawley rat MSCs were seeded on the surface of the wraps. We then compared the effects of five AVF treatments—no perivascular wrap (i.e., control), PCL wrap, PCL + MSC wrap, PCL-Au wrap, and PCL-Au + MSC wrap—on AVF maturation in a Sprague Dawley rat model of chronic kidney disease (n = 3 per group). Via micro-CT, AuNP-infused wraps demonstrated a significantly higher radiopacity compared to that of the wraps without AuNPs. Wraps with and without AuNPs equally reduced vascular stenoses, as seen via ultrasonography and histomorphometry. In the immunofluorescence analysis, representative MSC-seeded wraps demonstrated reduced neointimal staining for markers of infiltration with smooth muscle cells (α-SMA), inflammatory cells (CD45), and fibroblasts (vimentin) compared to that of the control and wraps without MSCs. In conclusion, AuNP infusion allows in vivo monitoring via micro-CT of MSC-seeded polymeric wraps over time, without compromising the benefits of the wrap for AVF maturation.

## 1. Introduction

Approximately 2.3 million individuals are undergoing chronic hemodialysis worldwide, and a majority of them rely on an arteriovenous fistulas (AVF) for vascular access [1]. In the United States, 62.6% of patients with a prevalent end-stage renal disease depend on an AVFs for hemodialysis [2]. Although an AVF is preferred over alternative forms of vascular access (i.e., intravenous catheter or arteriovenous graft) because of the lower rates of infection and maintenance interventions [3], its utility is still limited by high rates of maturation failure [4,5,6]. One of the leading causes of AVF failure is neointimal hyperplasia (NIH), a cellular process brought on by the synergistic action of hemodynamic stress, inflammation, and hypoxia on the vascular wall [7,8]. Although most investigational systemic therapies have failed to demonstrate a clear benefit in improving AVF maturation rates, a wide array of localized perivascular interventions, such as mechanical devices and cell-based therapies, have shown potential in mitigating NIH and AVF failures [9].

In a recent study, the perivascular application of mesenchymal stem cell (MSC)-seeded polymeric scaffolds in the form of wraps sutured around the AVF was shown to reduce NIH and enhance AVF maturation [10]. However, the polymeric wraps’ radiolucency makes in vivo monitoring challenging. In vivo monitoring is an essential component of medical device surveillance, and in its absence, the safety and efficacy of a medical device are both compromised. Although wraps are clearly delineated from the surrounding vascular layers in histology testing, they are not easily delineated on in vivo imaging (i.e., ultrasonography (US) and ^18^F-FDG PET). As a solution, polymeric constructs can be synthesized with metallic nanoparticles to give them radiopacity that will enable the tracking of their placement, integrity, and breakdown over time [11,12]. Among the currently available metallic nanoparticles, gold nanoparticles (AuNPs) have shown an excellent tunability, radiopacity, and biocompatibility [13,14].

In this study, we have synthesized an electrospun polymeric perivascular wrap from AuNP-infused polycaprolactone (PCL), seeded it with MSCs, and compared its effect on AVF maturation against that of non-AuNP-containing wraps. Additionally, this study aimed to monitor the effect of polymeric wraps on the AVF outflow vein for a longer period of observation (i.e., 8 weeks) than previously reported, with an initial assessment in as early as 2 weeks. We hypothesized that the incorporation of AuNPs would confer sufficient radiopacity to an MSC-seeded polymeric wrap, as seen via micro-CT, without affecting the wrap’s beneficial effects on the outflow vein of the AVF.

## 2. Results

### 2.1. Physicochemical Characteristics of the Polymeric Wraps

**Figure 1:**
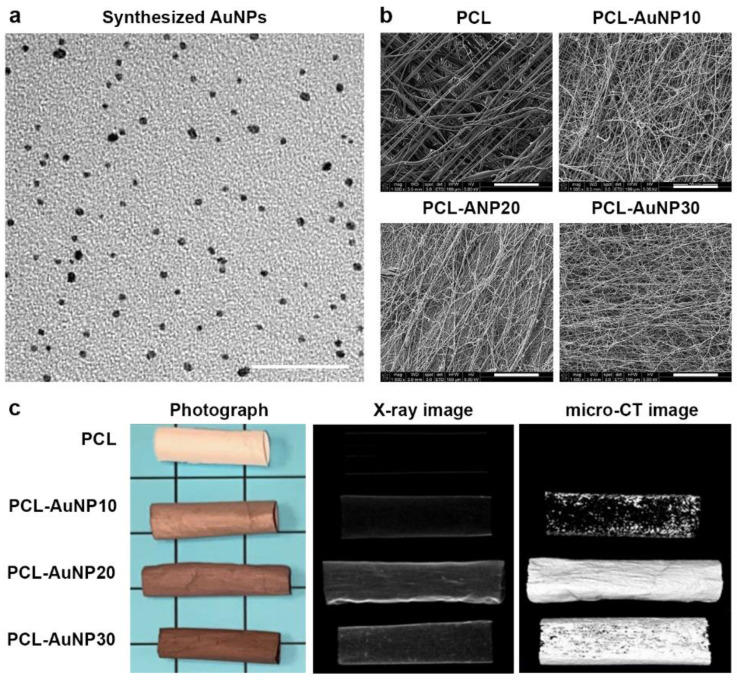
Representative images of the polymeric wraps. (**a**) Transmission electron microscopy image of synthesized gold nanoparticles (AuNPs) (bar = 50 μm). (**b**) Representative scanning electron microscopy images of the polymeric wraps (bars = 50 μm). (**c**) Representative photographic, X-ray, and micro-CT images of the polymeric wraps. Abbreviation: PCL, polycaprolactone.

**Figure 2:**
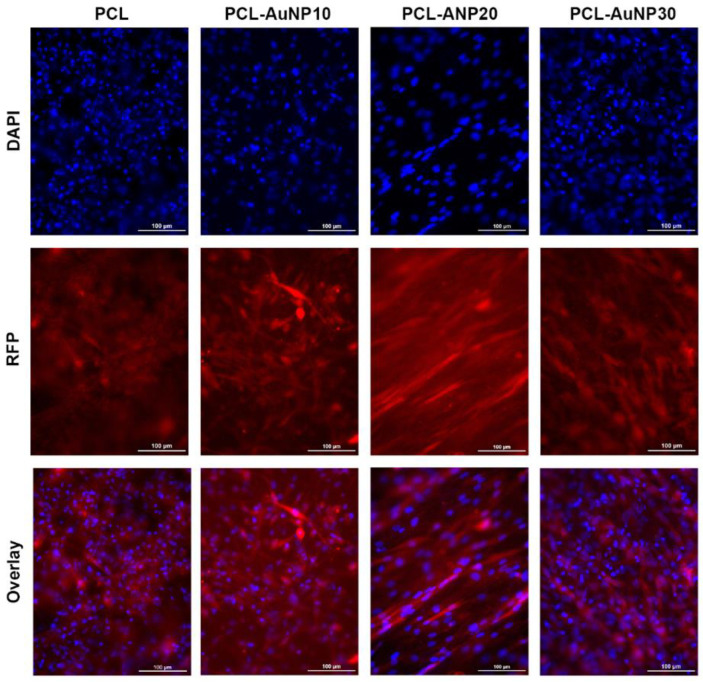
Attachment of Sprague Dawley rat red fluorescent protein (RFP)-expressing mesenchymal stem cells (MSCs) onto the polymeric wraps. The red areas represent the RFP-stained cytoplasm, while the blue dots represent the nuclear counterstain with DAPI. All the polymeric wraps allowed sufficient attachment of Sprague Dawley rat RFP-MSCs after 48 h of incubation (bars = 100 μm). Abbreviations: AuNP, gold nanoparticle; DAPI, 4′,6-diamidino-2-phenylindole; PCL, polycaprolactone.

Figure 1 shows a representative TEM image of the synthesized AuNPs as well as representative SEM, photographic, X-ray, and micro-CT images of the electrospun polymeric wraps. The synthesized AuNPs had an average diameter of 3.93 ± 0.68 nm. The physicochemical properties of the electrospun polymeric wraps are summarized in Table 1. These properties translated to the adequate attachment of MSCs, as seen via fluorescence microscopy (Figure 2). Among the AuNP-infused wraps, PCL-AuNP20 (20% concentration of AuNPs) demonstrated a qualitatively more consistent signal intensity on both the X-ray and micro-CT images. Hence, this blend was chosen for subsequent experiments, termed hereafter as “PCL-Au”.

### 2.2. AuNP-Infused Wraps Demonstrated Higher Radiopacity on Micro-CT Images

**Figure 3:**
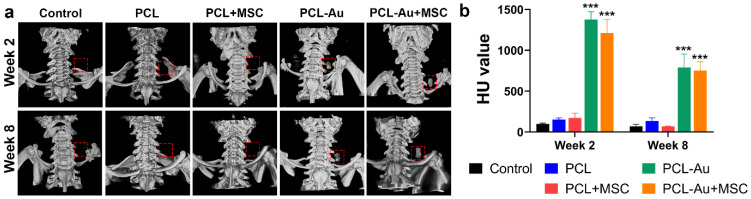
Evaluation of radiopacity via micro-CT. (**a**) Reconstructed micro-CT images of the control (i.e., no perivascular wrap), PCL, PCL + MSC, PCL-Au, and PCL-Au + MSC arteriovenous fistulas (AVFs) at 2 and 8 weeks after AVF creation. Red boxes correspond to the position of the wrap. (**b**) HU values of the five groups of AVFs 2 and 8 weeks after AVF creation; at weeks 2 and 8, rat AVFs wrapped with AuNP-containing polymeric scaffolds (PCL-Au and PCL-Au + MSC) had higher HU values compared to those of the control (*p* < 0.001) and non–Au-containing wraps (PCL and PCL + MSC; both *p* < 0.001). Abbreviations: ***, *p* < 0.001; Au, gold; HU, Hounsfield unit; PCL, polycaprolactone; MSC, mesenchymal stem cell.

Figure 3 and Table 2 compare the micro-CT findings for the five groups of rat AVFs 2 and 8 weeks after AVF creation.

In week 2, a higher radiopacity was seen in the rat AVFs wrapped with polymeric wraps containing AuNPs (PCL-Au and PCL-Au + MSC) compared to those of the control (*p* < 0.001) and non-Au containing wraps (PCL (*p* < 0.001) and PCL + MSC (*p* < 0.001)). There was no difference among the AVFs without AuNPs, i.e., between control and PCL (*p* = 0.95), control and PCL + MSC (*p* = 0.85), and PCL and PCL + MSC (*p* > 0.99). There was also no difference between the PCL-Au and PCL-Au + MSC (*p* = 0.21) AVFs.

In week 8, the radiopacity of AuNP-containing wraps was retained. PCL-Au and PCL-Au + MSC AVFs still had higher radiopacity compared to those of the control (*p* < 0.001), PCL (*p* < 0.001), and PCL + MSC (*p* < 0.001) AVFs. Similar to week 2, there was no difference between the control and PCL (*p* = 0.89), control and PCL + MSC (p > 0.99), or PCL and PCL + MSC (*p* = 0.88) AVFs. There was also no difference between the PCL-Au and PCL-Au + MSC (*p* = 0.98) AVFs. However, there was a larger reduction in the HU values of the PCL-Au (42.54%, *p* < 0.001) and PCL-Au + MSC (37.96%, *p* = 0.002) wraps in week 8 as compared to that in week 2. Nonetheless, the intensity level of the PCL-Au and PCL-Au + MSC wraps remained similar (*p* = 0.98), which suggests that the addition of MSCs did not affect the wraps’ degradation over time.

### 2.3. AuNP-Infused and Non-AuNP Wraps Equally Improved the Wall-to-Lumen Ratio of the Outflow Vein

**Figure 4:**
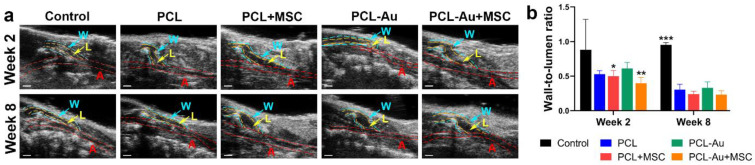
Evaluation of wall-to-lumen ratio via B-mode US. (**a**) B-mode US images of the control (i.e., no perivascular wrap), PCL, PCL + MSC, PCL-Au, and PCL-Au + MSC arteriovenous fistulas (AVFs) at 2 and 8 weeks after AVF creation (A, artery (red); L, lumen of outflow vein (yellow); W, wall of outflow vein (blue); bars = 1 mm). (**b**) Wall-to-lumen ratio of the five groups of AVFs 2 and 8 weeks after AVF creation; at week 2, PCL + MSC (*p* = 0.045) and PCL-Au + MSC (*p* = 0.008) AVFs had a lower wall-to-lumen ratio compared to that of the control, and at week 8, all scaffold groups had a lower wall-to-lumen ratio compared with the that of the control rat AVFs (*p* < 0.001). Abbreviations: *, *p* < 0.05; **, *p* < 0.002; ***, *p* < 0.001; Au, gold; PCL, polycaprolactone; MSC, mesenchymal stem cell.

We assessed the wall-to-lumen ratio as a marker of the vascular stenosis. Figure 4 and Table 2 compare the US findings for the five groups of rat AVFs at 2 and 8 weeks after AVF creation. We discovered that the PCL + MSC and PCL-Au + MSC AVFs had a more reduced wall-to-lumen ratio, a marker of vascular stenosis, in comparison to that of the AVFs without MSCs as early as 2 weeks after AVF creation.

In week 2, the control rat AVFs had a higher wall-to-lumen ratio compared to that of the PCL + MSC (*p* = 0.045) and PCL-Au + MSC (*p* = 0.008) AVFs. There was no difference between the control and PCL (*p* = 0.072), control and PCL-Au (*p* = 0.24), PCL and PCL + MSC (*p* > 0.99), PCL and PCL-Au (*p* = 0.96), PCL and PCL-Au + MSC (*p* = 0.83), PCL + MSC and PCL-Au (*p* = 0.90), PCL + MSC and PCL-Au + MSC (*p* = 0.92), or PCL-Au and PCL-Au + MSC (*p* = 0.45) AVFs.

In week 8, the control rat AVFs had a higher wall-to-lumen ratio than all the other wrap groups did: PCL (*p* < 0.001), PCL + MSC (*p* < 0.001), PCL-Au (*p* < 0.001), and PCL-Au + MSC (*p* < 0.001). There was no difference between the PCL and PCL + MSC (*p* = 0.99), PCL and PCL-Au (*p* > 0.99), PCL and PCL-Au + MSC (*p* = 0.98), PCL + MSC and PCL-Au (*p* > 0.95), PCL + MSC and PCL-Au + MSC (*p* > 0.99), or PCL-Au and PCL-Au + MSC (*p* = 0.94) AVFs. The similarity between the wall-to-lumen ratios of PCL + MSC and PCL-Au + MSC AVFs suggests that the incorporation of AuNPs does not affect the efficacy of MSCs in reducing the AVF wall-to-lumen ratio.

### 2.4. AuNP-Infused and Non-AuNP Wraps Equally Reduced NIH at the Outflow Vein

**Figure 5:**
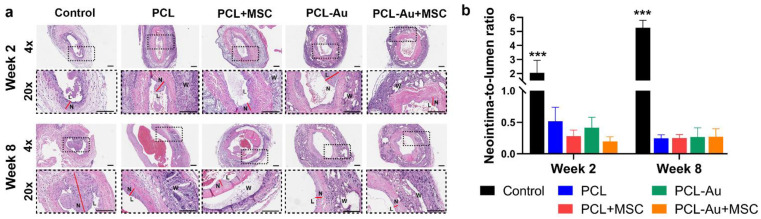
Evaluation of neointima-to-lumen ratio via histomorphometric analysis. (**a**) Hematoxylin and eosin-stained microscopic images of the control (i.e., no perivascular wrap), PCL, PCL + MSC, PCL-Au, and PCL-Au + MSC arteriovenous fistulas (AVFs) at 2 and 8 weeks after AVF creation (L, lumen; N, neointima; W, wrap; bars = 200 μm). Dashed boxes in 4× images show the areas of 20× magnification. Red lines indicate areas of neointimal hyperplasia (N). Red blood cells within the lumen of the vessels resulting from incomplete tissue processing were excluded from the analysis. (**b**) Neointima-to-lumen ratio of the five groups of AVFs 2 and 8 weeks after AVF creation; at weeks 2 and 8, the control rat AVFs had a higher neointima-to-lumen ratio compared to all that of the other groups. Abbreviations: ***, *p* < 0.001; Au, gold; PCL, polycaprolactone; MSC, mesenchymal stem cell.

We assessed the neointima-to-lumen ratio to assess the presence of NIH. Figure 5 and Table 2 compare the histomorphometric analyses of the five groups of rat AVFs at 2 and 8 weeks after AVF creation. We found that 8 weeks after AVF creation, all the treatment groups had improved neointima-to-lumen ratios compared to those of the control rats.

In week 2, the control rat AVFs had a higher neointima-to-lumen ratio compared to that of all the other groups: PCL (*p* < 0.001), PCL + MSC (*p* < 0.001), PCL-Au (*p* < 0.001), and PCL-Au + MSC (*p* < 0.001). There was no difference between the PCL and PCL + MSC (*p* = 0.92), PCL and PCL-Au (*p* = 0.97), PCL and PCL-Au + MSC (*p* = 0.81), PCL + MSC and PCL-Au (*p* > 0.99), PCL + MSC and PCL-Au + MSC (*p* > 0.99), or PCL-Au and PCL-Au + MSC (*p* = 0.99) AVFs.

In week 8, the control rat AVFs maintained a higher neointima-to-lumen ratio compared to that of all the other groups: PCL (*p* = 0.001), PCL + MSC (*p* = 0.002), PCL-Au (*p* = 0.002), and PCL-Au + MSC (*p* < 0.001). There was no difference between the PCL and PCL + MSC (*p* = 0.93), PCL and PCL-Au (*p* = 0.95), PCL and PCL-Au + MSC (*p* > 0.99), PCL + MSC and PCL-Au (*p* > 0.99), PCL + MSC and PCL-Au + MSC (*p* = 0.94), or PCL-Au and PCL-Au + MSC (*p* = 0.96) AVFs. Similar to what was observed with the wall-to-lumen ratio, the addition of AuNPs did not influence the wraps’ capacity to reduce the AVF neointima-to-lumen ratio.

### 2.5. MSC-Seeded Wraps Reduced Neointimal α-SMA, CD45, and Vimentin Staining

**Figure 6:**
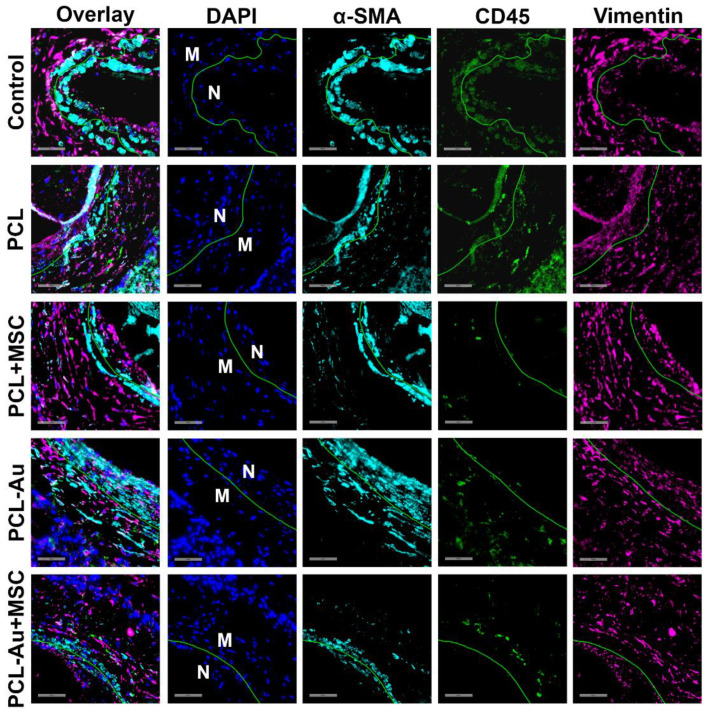
Evaluation of neointimal immunofluorescence staining for α-SMA, CD45, and vimentin. Immunofluorescence microscopic images of the control (i.e., no perivascular wrap), PCL, PCL + MSC, PCL-Au, and PCL-Au + MSC arteriovenous fistulas (AVFs) at 8 weeks after AVF creation, showing the different cell populations in the neointimal (N) and medial (M) vascular layers (bars = 50 μm). The green line marks the separation between the neointimal (N) and medial (M) layers. The following stains are depicted: α-SMA (teal), CD45 (green), vimentin (pink), and DAPI (blue). Abbreviations: Au, gold; α-SMA, alpha-smooth muscle actin; CD, cluster of differentiation; DAPI, 4′,6-diamidino-2-phenylindole; MSC, mesenchymal stem cell; PCL, polycaprolactone.

We assessed several markers that represent neointimal infiltration with smooth muscle cells, inflammatory cells, and fibroblasts (i.e., α-SMA, CD45, and vimentin, respectively). Table 3 and Figure 6 compare the immunofluorescence analysis results of representative slides from each of the five groups of rat AVFs at 8 weeks after AVF creation. When compared to the control, the PCL and PCL-Au wraps reduced the neointima-to-media ratio of the total cells (i.e., DAPI), as well as the CD45- and vimentin-positive cells, but increased the neointima-to-media ratio of the α-SMA-positive cells. In contrast, PCL + MSC and PCL-Au + MSC wraps reduced the neointima-to-media ratio of α-SMA-positive cells, and these MSC-seeded wraps further decreased the neointima-to-media ratio of the total cells, as well as CD45- and vimentin-positive cells when compared to that of their non-MSC counterparts.

## 3. Discussion

Prior studies have shown that a mesenchymal stem cell (MSC)-seeded polymeric wrap is capable of reducing neointimal hyperplasia (NIH) and enhancing arteriovenous fistula (AVF) maturation [10]. However, the radiolucent nature of the polymeric wrap makes the in vivo monitoring of its deployment and structural integrity difficult. To address this limitation, we incorporated AuNPs in the synthesis of polycaprolactone (PCL) wraps, which turned the wraps more radiopaque than their non-AuNP counterparts are, and consequently, are clearly observable via micro-CT at up to 8 weeks. This property is useful in AVF surveillance since it facilitates the quick localization of the AVF for concurrent studies using other imaging modalities, such as US or ^18^F-FDG PET. The integration of AuNPs within the architecture of the wraps facilitates the confirmation of device placement after surgery, and the reduction in the detected intensity from the wrap may serve as a marker of its degradation over time.

We found that PCL + MSC and PCL-Au + MSC AVFs demonstrated an improved wall-to-lumen ratio, a marker of vascular stenosis, compared with that of the AVFs without MSCs in as early as 2 weeks after AVF creation. This is consistent with the results of a prior study, wherein the addition of MSCs to a polymeric wrap reduced the AVF wall-to-lumen ratio compared to that of a polymeric wrap alone at 4 weeks after AVF creation [10]. These results highlight the unique role of MSCs in improving AVF maturation. The similarity between the wall-to-lumen ratios of PCL + MSC and PCL-Au + MSC AVFs (*p* = 0.92) also suggests that the incorporation of AuNPs does not affect the activity of MSCs in reducing the AVF wall-to-lumen ratio. By week 8, all the treatment groups demonstrated an improved wall-to-lumen ratio, but the MSCs no longer conferred an advantage. Nonetheless, the similarity between the wall-to-lumen ratios of AVFs with and without MSCs still supports the idea that the incorporation of AuNPs does not interfere with the activity of the wraps in reducing an AVF’s wall-to-lumen ratio.

We also found that all the treatment groups demonstrated improved neointima-to-lumen ratios, a measure of NIH, versus that of the control rats by 8 weeks after AVF creation. Our findings show that the reduction in the neointima-to-lumen ratio by the PCL and PCL + MSC treatments versus that of the control seen at 4 weeks after AVF creation in a previous study [10] can be seen as early as 2 weeks and can be maintained for up to 8 weeks. Moreover, the incorporation of AuNPs did not affect the ability of the wraps to reduce the AVF neointima-to-lumen ratio. We expected that the polymeric wraps alone could also reduce the stenosis of the venous outflow wall, since these wraps could attenuate hemodynamic stress, one of the major pathways that underlies NIH, by mitigating vascular overexpansion, turbulence, and endothelial injuries [8]. Although it appears that MSC-containing wraps no longer provide an added benefit by week 8, there could be changes in the cellular and extracellular composition of the vascular wall attributable to MSCs that are not reflected in the results of in vivo imaging. Interestingly, we found that representative MSC-seeded wraps performed better than the control and non-MSC counterparts did in reducing the neointima-to-media ratio of total cells, as well as α-SMA-, CD45-, and vimentin-positive cells via immunofluorescence analysis. Moreover, we observed an increase in the neointima-to-media ratio of α-SMA-positive cells with PCL and PCL-Au AVFs, which indicates the inward migration of smooth muscle cells within the neointima after longer periods of observation. Taken together, these findings support the hypothesis that the addition of MSCs may promote better outward vascular remodeling compared to that of a vascular wrap that serves purely as a physical support.

The scope of this study was limited to the assessment of the signal intensity of wraps via micro-CT and the effect of the wraps on the AVF wall-to-lumen ratio, as seen via US, and the neointima-to-lumen ratio, as seen via histology testing. Nonetheless, it represents a significant stride towards the development of an all-in-one electrospun perivascular wrap that can be non-invasively monitored, while providing both mechanical and functional support to the maturing AVF. The use of cells that can potentially suppress NIH, such as endothelial cells and mesenchymal stem/stromal cells, will continue to be an emerging area of active research since cell-based therapies could release a wider array of immunomodulatory substances for AVF maturation compared to those of localized perivascular drug delivery. Progress in this field can be accelerated through the development of new applications for known biocompatible materials, such as synthetic polymers and nanoparticles. Future studies are recommended to explore the relationship between the rate of wrap degradation and its impact on the ability of the wrap to provide attachment to seeded MSCs as well as mechanical support to the maturing AVF. Moreover, there is a need for additional research on the cellular and extracellular composition of the vascular wall in order to determine the fate of the seeded MSCs, understand the effect of MSCs on the different cells of the outflow vein, and identify potential sources of differences between in vivo and histological observations in this study. These investigations will pave the way for the application of this research to larger animals and, eventually, to human patients who may benefit from novel AVF therapies.

## 4. Materials and Methods

### 4.1. Synthesis of AuNP-Infused Polymeric Wraps

AuNPs were synthesized following a published protocol by Tian et al. [15]. AuNPs and PCL (average M_n_ 80,000, Sigma-Aldrich, St. Louis, MO, USA) were mixed with toluene/MeOH to form a solution with 15% PCL and increasing concentrations of AuNPs—0%, 10%, 20%, and 30%. The resulting solutions were electrospun at 1.0 mL/h and 15 kV using a Spraybase electrospinning system (Avectas, Maynooth, Ireland). The synthesis and characterization of AuNP-infused polymeric wraps, MSC culture and attachment processes, AVF creation and perivascular wrap application, US, and histological analysis were conducted as described in a prior study [10].

### 4.2. Characterization of AuNP-Infused Polymeric Wraps

The size and morphology of AuNPs were determined using a JEM 1010 transmission electron microscope (TEM, JEOL USA, Inc., Peabody, MA, USA). The fiber diameter and pore size of wraps were determined using a Nova NanoSEM scanning electron microscope (SEM, Field Electron and Ion Company, Hillsboro, OR, USA) with an EDAX energy-dispersive spectroscopy system (Ametek, Berwyn, PA, USA). SEM images were captured from random areas to determine the mean fiber diameter and mean pore size of each sample using ImageJ 1.54d software (National Institutes of Health, Bethesda, MD, USA). The porosity was determined using the calculation method [16] and liquid intrusion method [17]. The melting temperature (T_m_) and glass transition temperature (T_g_) were determined using an STA PT 1000 thermogravimetric analyzer (Linseis, Selb, Germany). Ultimate tensile strength was determined using an MTESTQuattro materials testing system (ADMET, Norwood, MA, USA).

### 4.3. Cell Culture and MSC Attachment

Bone-marrow-derived Sprague Dawley rat mesenchymal stem cells that express red fluorescent protein (RFP-MSCs, P2, Creative Bioarray, Shirley, NY, USA) were cultured in DMEM with 10% FBS and 1% penicillin–streptomycin (Corning, Corning, NY, USA). The wraps were subjected to ethylene oxide gas sterilization, and then seeded with 1 × 10^5^ RFP-MSCs. After 48 h, the samples were imaged using an Eclipse Ti2 fluorescence microscope (Nikon, Melville, NY, USA) to visualize the attachment of RFP-MSCs. The cells for in vivo testing were kept in culture media before surgical application.

### 4.4. AVF Creation and Perivascular Wrap Application

All rat experiments were approved by the Institutional Animal Care and Use Committee. We created a chronic kidney disease (CKD) model using Sprague Dawley rats (400 SAS SD, Charles River Laboratories, Wilmington, MA, USA) using the two-step 5/6th nephrectomy procedure by Wang et al. [18]. After 4 weeks, we then created an AVF model via the end-to-side surgical anastomosis of the external jugular vein with the common carotid artery based on a procedure by Wong et al. [19]. A total of 15 rats were randomly allocated into five groups—control AVF (i.e., no wrap), PCL wrap, PCL + MSC wrap, PCL-Au wrap, and PCL-Au + MSC wrap—with 3 rats per group. Based on our pilot data, a minimum of 3 rats per group is needed to attain a statistical level of *p* < 0.05 with a power of 80%. In the wrap groups, a 1 mm × 5 mm piece of the gas-sterilized cylindrical wrap was placed around the outflow vein and secured using 10-0 nylon sutures. The MSC groups received a wrap seeded with 1 × 10^5^ MSCs.

### 4.5. X-ray and Micro-CT

Prior to in vivo application, a Skyscan 1276 CMOS micro-CT system (Bruker, Billerica, MA, USA) was used to capture X-ray and micro-CT images of the wraps. The radiopacity of the implanted wraps was then determined using the same machine. The CTAn micro-CT image analyzer (Bruker) was used to measure the radiopacity of the wraps in Hounsfield units (HU) and perform volume rendering for three-dimensional visualization.

### 4.6. Ultrasonography

A Vevo 2100-LAZR system (FUJIFILM VisualSonics, Toronto, ON, Canada) was used to visualize pulsatile arterial waveforms in the external jugular vein of successfully created AVFs. At weeks 2 and 8, the rats were anesthetized, and a 15-MHz B-mode probe was used to obtain 6 measurements of the wall thickness and luminal diameter across the outflow vein. The wall-to-lumen ratio was calculated by dividing the average wall thickness by the average luminal diameter.

### 4.7. Histological Analysis

Rats were euthanized via CO_2_ asphyxiation and thoracotomy following imaging. The outflow veins were harvested after perfusion for formalin fixation and paraffin embedding. Tissue blocks were cut into 4 μm sections and subjected to hematoxylin and eosin (H&E) staining or immunofluorescence multiplex staining with the following markers: (1) alpha-smooth muscle actin (α-SMA), which is highly expressed in smooth muscle cells and also expressed in fibroblasts; (2) CD45, which is highly expressed in leukocytes and a marker of inflammatory infiltration; (3) vimentin, which is highly expressed in fibroblasts and also expressed in vascular smooth muscle cells and lymphocytes; and (4) 4′,6-diamidino-2-phenylindole (DAPI), a nuclear stain. An Aperio LV1 real-time digital pathology system (Leica Biosystems, Buffalo Grove, IL, USA) was used to capture images from the H&E slides and quantify the luminal and neointimal areas. Blood artifacts seen within the lumen of vessels resulting from incomplete tissue processing were excluded from the analysis. A Leica Versa fluorescent-imaging system (Leica Biosystems) was used to capture images from the immunofluorescent slides, and the Halo image analysis platform (Indica Labs, Albuquerque, NM, USA) was used to perform fluorescent cell counting.

### 4.8. Statistical Analysis

Quantitative data are presented as means ± standard deviations and analyzed using one-way analysis of variance with GraphPad Prism software, version 9.0.0 (GraphPad, San Diego, CA, USA). All rats were included in the analysis, and statistical significance was defined as *p* < 0.05.

## 5. Conclusions

Overall, our results indicate that AuNP infusion permits in vivo monitoring via micro-CT of the placement and integrity over time of an MSC-seeded polymeric wrap, while preserving the positive effects of the wrap on AVF maturation. Our findings also confirm the role of MSCs in promoting outward vascular remodeling in the setting of AVF maturation. In conclusion, this study provides a basis for the development and clinical use of an MSC-loaded AuNP-infused perivascular wrap to improve AVF maturation and patency.

## Figures and Tables

**Table 1:** Physicochemical properties of the electrospun polymeric wraps.

Wrap Type	Diameter (μm)	Pore Size (μm)	Porosity (Calculated, %)	Porosity (Intrusion, %)	T_g_ (°C)	T_m_ (°C)	Maximum Stress (MPa)
PCL	2.07 ± 1.03	168.34 ± 177.94	93.21 ± 1.23	76.75 ± 8.06	52.53	−69.61	9.17 ± 0.31
PCL-AuNP10	0.53 ± 0.26	98.34 ± 76.13	53.63 ± 20.62	74.31 ± 6.15	54.32	−68.19	13.41 ± 0.52
PCL-AuNP20	0.25 ± 0.07	81.57 ± 86.11	65.52 ± 1.13%	71.13 ± 1.98	52.44	−69.71	9.01 ± 0.42
PCL-AuNP30	0.27 ± 0.09	62.91 ± 53.40	64.02 ± 8.34	76.03 ± 3.69	52.37	−69.64	12.42 ± 1.40

Abbreviations: AuNP, gold nanoparticles; PCL, polycaprolactone; T_g_, glass transition temperature; T_m,_ melting temperature.

**Table 2:** Radiopacity, wall-to-lumen ratio, and neointima-to-lumen ratio of the AVF groups (n = 3 per group).

Wrap Type	Radiopacity (HU)		Wall-to-Lumen Ratio		Neointima-to-Lumen Ratio	
	Week 2	Week 8	Week 2	Week 8	Week 2	Week 8
Control	98.07 ± 9.88	69.27 ± 23.81	0.88 ± 0.44	0.95 ± 0.03	2.05 ± 0.89	5.26 ± 0.53
PCL	152.0 ± 21.21	135.8 ± 37.19	0.53 ± 0.05	0.31 ± 0.08	0.52 ± 0.22	0.25 ± 0.06
PCL + MSC	171.9 ± 60.22	67.68 ± 5.61	0.5 ± 0.08	0.24 ± 0.04	0.28 ± 0.10	0.25 ± 0.06
PCL-Au	1375 ± 97.79	790.1 ± 164.0	0.61 ± 0.09	0.33 ± 0.08	0.34 ± 0.24	0.27 ± 0.15
PCL-Au + MSC	1212 ± 164.1	751.9 ± 107.1	0.4 ± 0.09	0.23 ± 0.05	0.20 ± 0.08	0.27 ± 0.13

Abbreviations: Au, gold; AVF, arteriovenous fistula; HU, Hounsfield unit; MSC, mesenchymal stem cell; PCL, polycaprolactone.

**Table 3:** Neointima-to-media ratio of immunofluorescence-stained cells on representative AVFs.

Wrap Type	α-SMA	CD45	Vimentin	DAPI
Control	1.0	0.76	0.74	0.79
PCL	1.21	0.30	0.71	0.69
PCL + MSC	0.98	0.17	0.09	0.64
PCL-Au	1.74	0.08	0.36	0.66
PCL-Au + MSC	0.92	0.07	0.22	0.40

Abbreviations: α-SMA, alpha-smooth muscle actin; Au, gold; AVF, arteriovenous fistula; CD45, cluster of differentiation 45; DAPI, 4′,6-diamidino-2-phenylindole; MSC, mesenchymal stem cell; PCL, polycaprolactone.

## Data Availability

The datasets used and/or analyzed during the current study are available from the corresponding author upon reasonable request.

## Abbreviations

α-SMA	alpha smooth muscle actin
AuNP	gold nanoparticle
AVF	arteriovenous fistula
CD45	cluster of differentiation 45
CKD	chronic kidney disease
CT	computed tomography
DAPI	4′,6-diamidino-2-phenylindole
^18^F-FDG PET	^18^F-fluorodeoxyglucose positron emission tomography
H&E	hematoxylin and eosin
HU	Hounsfield units
MSC	mesenchymal stem cell
NIH	neointimal hyperplasia
PCL	polycaprolactone
RFP	red fluorescent protein
SEM	scanning electron microscope
TEM	transmission electron microscope
US	ultrasound
